# Social Feedback During Sensorimotor Synchronization Changes Salivary Oxytocin and Behavioral States

**DOI:** 10.3389/fpsyg.2020.531046

**Published:** 2020-09-23

**Authors:** Claudiu C. Papasteri, Alexandra Sofonea, Romina Boldasu, Cǎtǎlina Poalelungi, Miralena I. Tomescu, Constantin A. D. Pistol, Rǎzvan I. Vasilescu, Cǎtǎlin Nedelcea, Ioana R. Podina, Alexandru I. Berceanu, Robert C. Froemke, Ioana Carcea

**Affiliations:** ^1^CINETic Center, National University of Theatrical and Cinematographic Arts, Bucharest, Romania; ^2^Faculty of Psychology and Educational Sciences, Department of Psychology, University of Bucharest, Bucharest, Romania; ^3^Faculty of Biology, University of Bucharest, Bucharest, Romania; ^4^Faculty of Physics, Department of Electricity, Solid Physics and Biophysics, University of Bucharest, Bucharest, Romania; ^5^Skirball Institute, Department of Otolaryngology, New York University Grossman School of Medicine, New York, NY, United States; ^6^Skirball Institute, Department of Neuroscience and Physiology, New York University Grossman School of Medicine, New York, NY, United States; ^7^Brain Health Institute, Department of Pharmacology, Physiology and Neuroscience, New Jersey Medical School, Rutgers, The State University of New Jersey, Newark, NJ, United States

**Keywords:** oxytocin (OXT), social synchronization, social approval, closeness, well-being, gender, personality traits

## Abstract

In humans and animal models, oxytocin increases social closeness, attachment and prosocial behaviors, while decreasing anxiety and stress levels. Efficiently triggering the release of endogenous oxytocin could serve as a powerful therapeutic intervention for disorders of social behavior and for anxiety. We designed a new version of a social sensorimotor synchronization task to investigate the role of social approval in inducing biochemical and psychological changes following behavioral synchrony in a sample of 80 college students. Social approval in the form of real time positive feedback increased well-being only in women, while increasing social closeness in both genders. Social disapproval in the form of real time negative feedback prevented a decrease in stress levels that otherwise women reported following engagement in either social or non-social synchronization. Surprisingly, for certain personality traits, negative social feedback during sensorimotor synchronization was psychologically beneficial irrespective of gender. Salivary oxytocin levels increased only in women after the social but not the non-social synchronization tasks. Oxytocin dynamics were independent of the type of real time feedback that subjects received, indicating the existence of distinct mechanisms for hormonal versus behavioral changes following synchronization. Nevertheless, changes in salivary oxytocin after positive social feedback correlated with changes in well-being and predicted changes in prosocial attitudes. Our findings show evidence of distinct mechanisms for behavioral versus hormonal changes following social sensorimotor synchronization, and indicate that gender and personality traits should be carefully considered when designing behavioral therapies for improving social attitudes and for stress management.

## Introduction

The neuropeptide oxytocin (OXT) plays complex roles in emotional and social behaviors, including attachment, social recognition, trust, generosity, anxiety management, and responses to social stress (Heinrichs et al., 2003; Kosfeld et al., 2005; Uvnäs-Moberg et al., 2005; Zak et al., 2007; De Dreu et al., 2010; MacDonald and Feifel, 2014). OXT administration has been proposed as a treatment option for autism, social anxiety and other disorders with a social dimension (Andari et al., 2018; Benner and Yamasue, 2018). The excitement for the therapeutic potential of OXT has been tampered mostly by the difficulty to deliver the exogenous peptide to the brain, even when administered intranasally (Leng and Ludwig, 2016). Alternative approaches include behavioral therapies that stimulate the release of endogenous OXT. In addition to labor and lactation, OXT can be released after physical effort, sexual activity and physical touch (Seltzer et al., 2010; Jong et al., 2015). Recently, it has been shown that social imitation by sensorimotor synchronization can also increase OXT levels in dyadic partners (Aoki and Yamasue, 2015; Spengler et al., 2017).

Synchronized movements in dyadic social interactions were found to augment emotional expressiveness (Spengler et al., 2017), to improve transmission of emotional information (Aoki et al., 2014), to elicit heightened feelings of closeness to other (Ashton-James et al., 2007; Catmur and Heyes, 2013), greater generosity toward dyadic partner (Van Baaren et al., 2003), greater likeability of the other person (Hove and Risen, 2009), and generally more connectedness with them (Miles et al., 2009). Striking similarities between the reported effects of exogenous OXT with those of social imitation led to the hypothesis that social imitation may act on behavior through enhanced secretion of endogenous OXT (Aoki and Yamasue, 2015; Spengler et al., 2017). If this were indeed the case, we would expect that changes in endogenous OXT mediate behavioral changes, and that imitation leads to another major OXT effect on behavioral state, namely decreased anxiety or stress, and an increased sense of well-being. These aspects have not been investigated prior to our study.

Most of the behavioral and biochemical effects of social imitation have been investigated in the imitated partner (*imitee*) and less so in the *imitator*. This bias stemmed from the hypothesis that being imitated is critical for the emergence of attachment and bonding in young and adult human and non-human primates (Nadel-Brulfert and Baudonniere, 1982; Paukner et al., 2009; Hale and Hamilton, 2016).

### Current Study

Here, we investigated how social sensorimotor synchronization could be leveraged to become most beneficial for therapeutic interventions, in particular for improving stress-related behavioral states, for increasing social closeness and prosocial behaviors, and for increasing OXT levels. Although previous studies centered on the effects of social sensorimotor synchronization in the *imitee* (Aoki and Yamasue, 2015; Hale and Hamilton, 2016; Spengler et al., 2017), in our study we focused instead on the effects of sensorimotor synchronization on *imitators*. The rationale for this distinct approach stemmed from a pilot study where we compared the fluency of interactions when subjects were asked to initiate movements, versus when they were asked to follow the movements of an instructor. We found that subjects more easily engaged in experiments as followers than as initiators (data not shown). We reasoned then that participation in social sensorimotor synchrony as *imitator* would be more inclusive, permitting participation of socially anxious subjects, perhaps the category of people most likely to need such interventions. We tested the hypothesis that social sensorimotor synchronization can induce similar changes in behavioral states and in OXT levels in *imitators* as previously reported in *imitees*.

To increase the chances that *imitators* benefit from social sensorimotor synchronization, we reviewed the proposed mechanisms by which such interactions become profoundly beneficial in *imitees* (Hale and Hamilton, 2016). Several proximate mechanisms by which imitation and mimicry could impact behavioral states have been proposed (Hale and Hamilton, 2016). Generally, it is believed that detecting motor, cognitive (e.g., social conformity) and emotional alignment (e.g., emotional contagion) with others, indicates social approval to the *imitee*, which would be rewarding (Hale and Hamilton, 2016). We found particularly compelling this model which suggests that “being copied” by someone is rewarding (Kühn et al., 2010; Kokal et al., 2011; Hale and Hamilton, 2016), where social approval becomes reinforcing (Paukner et al., 2009). However, the role of social approval in inducing the behavioral effects of sensorimotor synchronization has not been tested directly. We hypothesized that directing social approval toward the *imitator* in the form of positive feedback might lead to similar behavioral and hormonal changes as in the *imitee*. To determine if perceived social approval might be the main driver of such profound changes following social sensorimotor synchronization, we investigated responses in *imitators* when they received social approval (positive feedback, “social positive” condition), social disapproval (negative feedback, “social negative” condition), or when they did not receive social feedback (“non-social” condition).

Personality traits, gender, and mental health may moderate the effects of imitation on endogenous OXT levels and on behavioral states, and should be considered when implementing oxytocinergic interventions (Bartz et al., 2011; Quirin et al., 2014). It remains unknown how gender and different personality traits might affect the biochemical and behavioral responses to synchronized social interactions. We examined the potentially moderating role of gender and of personality factors. We propose that social sensorimotor synchronization can be adjusted based on the gender and individual psychological profile of the subject, in order to produce most effective outcomes.

We tested several specific hypotheses: imitating others while receiving social approval cues increases salivary OXT, social closeness and well-being, and decreases stress; non-social sensorimotor synchronization and imitating others while receiving social disapproval cues do not change salivary OXT, social closeness, well-being, and stress; women have stronger responses than men to social sensorimotor synchronization with social approval cues. We also explored the possibility that changes in salivary OXT mediate changes in well-being and in social closeness, the possibility that social approval or disapproval during imitation changes prosocial attitudes, and the possibility that different personality types respond differently to imitation with social feedback.

## Materials and Methods

### Ethics Statement

All procedures applied in the present studies were accepted by the Ethics Committee of the National University of Theatrical and Cinematographic Arts in Bucharest, Romania, and were in accordance with the 1964 Helsinki declaration and its later amendments or comparable ethical standards. Informed consent was obtained from all individual participants included in the study.

### Participants

We recruited 80 somatic and mental healthy adults, aged between 18 and 40. Among these, 49 were women. The age of women (26.61 ± 0.89 years) was not different from that of men (26.65 ± 0.9) (Supplementary Figure S1).

### Procedures

Participants came to the laboratory on two different days with at least 1 week and at most 10 days in between. Experiments took place between noon and 16:00 local time, in a controlled environment with constant lighting, 25°C ambient temperature and sound protection. Before the experiment, participants were screened for depression (i.e., the Beck Depression Inventory-II; BDI-II test) and anxiety (i.e., the State Trait Anxiety Inventory-Y; STAI-Y test), and completed a personality inventory (i.e., NEO Personality Inventory – Revised; NEO PI-R). Before and after each experimental and control task, they completed several other self-report measures (described below). All subjects participated in the experimental social sensorimotor synchronization task with social approval (“social positive”), where they were asked to imitate a set of stereotyped, geometrical arm movements produced by an instructor (e.g., draw a circle in the air). The task time lasted for 6 min, during which time the instructor provided verbal and gestural positive feedback (e.g., enthusiastic “very good,” nodding yes, smiling). For control conditions, some of the subjects (49) participated in a non-social sensorimotor synchronization task (“non-social”), where they were asked to imitate the geometrical movements displayed on a screen, without social interactions. The rest of the subjects participated in a social synchronization task where the instructor provided negative verbal and gestural feedback (“social negative”) (e.g., “it’s not right, you are going to fast/slow,” sighing, frowning). The order of experimental and control task was randomized across subjects and counterbalanced. Supplementary Figure S2 presents a diagram of the experimental procedure.

### Self-Reported Measures

The inclusion of other in the self (IOS) (Aron et al., 1992) is a single-item, pictorial measure of *closeness*. The IOS is comprised of a series of 7 Venn-like diagrams, each of which is composed of two circles varying in their degree of overlap. No overlap (IOS = 1) indicates minimal subjective closeness, and maximum overlap (IOS = 7) indicates highest subjective closeness. In our studies, participants were instructed to select the diagram that best represents their relationships with the person in a black and white picture. This person was the instructor that guided the participants throughout the interventions. In 15 cases, the responses on the IOS scale were uninterpretable, and we subsequently removed them from the analysis. The acute emotional perception of *stress* was assessed using a 10-cm unmarked, description-anchored, visual analog scale (VAS), ranging from 0 (“no stress”) to 100 (“most stressed ever”). Momentary well-being was also measured on a 10-cm unmarked VAS ranging from 0 (worst imaginable) to 100 (perfect well-being).

The NEO Personality Inventory – Revised (NEO PI-R) (Costa and McCrae, 1992) is a 240-item *personality* inventory with cross-culturally established psychometric properties and validity (McCrae and Terracciano, 2005). The instrument assesses the Big Five Model domains of Neuroticism (N), Extraversion (E), Openness to experience (O), Agreeableness (A), and Conscientiousness (C), with six facets comprising each domain. Participants respond on a 5-point Likert scale ranging from 0 (strongly disagree) to 4 (strongly agree). Our studies employed the Romanian validated NEO PI-R (Costa et al., 2008). The cohort of subjects we included in the experiment were largely representative of the normal distribution of scores for personality traits (Supplementary Figure S3).

Depression Inventory II (BDI-II) (Beck and Steer, 1987; BDI-II; Beck et al., 1996) is a widely accepted 21-item self-report instrument used for the screening and assessment of *depression* in clinical and research settings. Respondents are asked to rate items on a 4-point Likert scale ranging from 0 to 3 based on severity of each item. Higher total scores indicate more severe depressive symptoms, with the maximal total score being 63. Our studies employed the Romanian validated BDI-II (Beck et al., 2012). The State-Trait Anxiety Inventory – Form Y (STAI-Y) (Spielberger, 1983; Spielberger, 1989) is a self-reported measure of anxiety with 40 items grouped in two scales: State (S-Anxiety) and Trait (T-Anxiety). The participants answered on a 4-point Likert scale, with higher scores indicating increased anxiety. The questionnaire has been shown to poses excellent psychometric properties for both the original English version (Barnes et al., 2002), as well as the Romanian adaptation (Spielberger, 2007), which was utilized in our study.

### Computerized Tasks

We used a computerized version of the Dictator Game (DG) which followed the procedure set by Brocklebank et al. (2011). We extracted our DG trials from Charness and Rabin’s (2002). Participants were informed that they will be playing with another anonymous player and that they will be alternating randomly between “Player A” and “Player B” roles. Player A (the dictator) receives an endowment (fixed quantity of virtual money) and gets to decide how much of this initial sum he/she would share with Player B. Player B can make no endowment and can only accept the amount offered by Player A. We used four DG trials and one sham trial. In all four DG trials, the participant was in a Player A role, while in the sham trial, the participant was in a Player B role. Participants were not aware of the total number of trials, when their roles would change or that they were playing against a fictitious character.

Each DG trial included a list of seven forced-choice alternatives of splitting the endowment on a continuum varying from altruistic (i.e., Player A chooses to remain with less virtual money than his/her counterplayer) to less altruistic choices (i.e., Player A chooses to remain with equal or less virtual money than his/her counterplayer). The sham trial was set to always offer the participant (now Player B) an equal amount of virtual money to that of his/her counterplayer so as not to prime in any manner the participant’s subsequent decisions as Player A.

Overall, the more amount of virtual money the participant decided not to share, the less altruistic he or she was in the game. The participant could remain throughout the whole four DG trials with an aggregated sum varying from 0 (i.e., Player A chooses to share all virtual money) to 900 (i.e., Player A chooses to share nothing). Notably, to increase motivation, participants were informed that they will receive a monetary reward which will be 1% of their virtual money choice in the game.

### Endocrine Measures

Salivary OXT level was measured two times a day. Participants provided saliva samples 15 min prior and 15 min after the experimental conditions as assisted by a qualified medical nurse. The saliva samples were collected in special designed tubes (Salivette^®^, Sarstedt). The participants were instructed to move the synthetic swab inside the salivette slowly in their mouth until it was saturated with saliva. The swab was then placed back into the specific tube and sent to the laboratory. There, the tubes were centrifuged at 1,000 × *g*, at 4°C, for 2 min and the samples were aliquoted in 1,5 ml Eppendorf vials and stored at −80°C prior to analysis. OXT was measured by radioimmunoassay (RIA) at RIAgnosis, Munich, Germany, while total proteins were measured at National Institute of Endocrinology “C. I. Parhon,” Bucharest, Romania. Measurements were done in two batches: the first batch had samples collected from the 30 subjects undergoing “social positive” and “social negative” conditions; the second batch had samples collected from the 50 subjects undergoing “social positive” and “non-social” conditions. Salivary total protein was used to normalize the concentration of salivary OXT levels, since its concentration can vary significantly with saliva viscosity. Five saliva probes were compromised in the processing and subsequently excluded from the analysis.

### Statistics

We investigated whether the post-intervention means, adjusted for pre-intervention scores, differ between experimental conditions using the ANCOVA method. The adjustment for the pre-intervention score in ANCOVA has two benefits. One is to make sure that any post-intervention differences truly result from the intervention and are not left-over effects of (usually random) pre-intervention differences between the groups. The other is to account for variation around the post-intervention means that comes from the variation in participant scores at pre-intervention.

As neither OXT nor behavioral data passed the D’Agostino & Pearson normality tests (alpha = 0.05), the analyses detailed in section “Results” employed the Wilcoxon matched-pairs signed rank test to assess pre- and post-intervention differences within the experimental conditions. The outcomes of these tests are summarized in Supplementary Table S1 (in Supplementary Material).

Exploratory analyses of relevant psychological characteristics as predictors of change were carried out through multiple regression. The goal was to assess the relationships of variables like personality traits, depression, and anxiety to direction and degree of change in outcome scores across time. Consequently, dynamic (change) outcome scores were introduced as the dependent variable, while pre-intervention scores were included as a covariates alongside the predictor variable inside the regression models.

To examine indirect effects of time on outcome change scores via changes in closeness to the interaction partner, we applied a within-participant mediation framework (Judd et al., 2001; Yzerbyt et al., 2018). This mediation analysis implies two steps. First, an examination of the component paths by means of joint significance test should find all component paths of the indirect effect significant in order to conclude in favor of mediation. Second, the magnitude and confidence interval of indirect effect is estimated by means of Monte Carlo resampling.

## Results

### Social Sensorimotor Synchronization Increases Salivary OXT

To determine if the social interactive aspect of engaging in sensorimotor imitation is indeed required for increasing endogenous OXT, we measured salivary OXT levels in all three conditions in the experiment. This allowed us to maintain a similar level of task engagement and an equivalent amount of movement between the social procedures and the “non-social” condition, isolating the contribution of the social factor to measured biochemical changes. We used a 2-session, 2-treatment crossover design.

**FIGURE 1:**
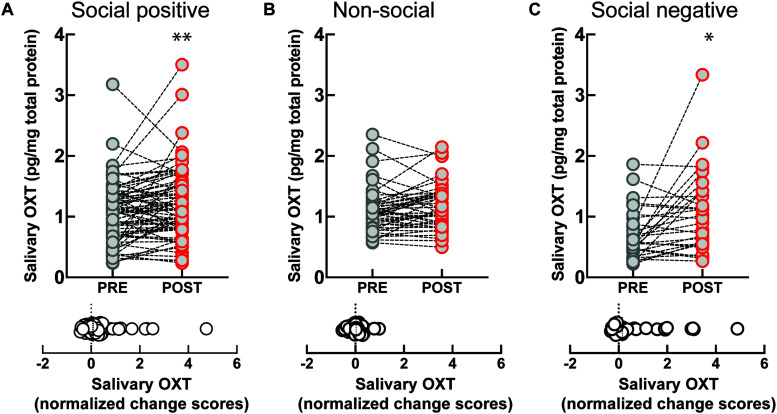
Effect of sensoriomotor imitation on endogenous OXT concentrations: **(A)** “social positive” condition, **(B)** “non-social” condition, and **(C)** “social negative” condition. OXT, oxytocin; PRE, before imitation; POST, after imitation. *p < 0.05, **p < 0.001.

The sample that engaged in the “social positive” task showed a significant increase in salivary OXT levels (PRE: 1.06 ± 0.05 pg/mg, POST: 1.20 ± 0.06 pg/mg, Wilcoxon matched-pairs signed rank test, *p* = 0.008, *N* = 75) (Figure 1A, top). On the contrary, no significant change was measured in the “non-social” condition (PRE: 1.09 ± 0.05 pg/mg, POST: 1.17 ± 0.05 pg/mg, *p* = 0.082, *N* = 46) (Figure 1B, top). To determine if the quality of social feedback during imitation was important for the observed biochemical changes, we measured salivary OXT before and after social imitation with negative feedback, “social negative.” In this paradigm, imitation still significantly increases OXT levels (PRE: 0.70 ± 0.07 pg/mg, POST: 1.07 ± 0.12 pg/mg, *p* = 0.010, *N* = 29) (Figure 1C, top). An ANCOVA with pre-intervention measure entered as a covariate to control for individual differences, confirmed that there was no significant difference between positive and negative feedback effects [*F*(1, 55) = 0.03, *p* = 0.852, $\eta_{p}^{2}$ = 0.01]. Similar results were obtained when we calculated baseline-normalized change scores [formula: (POST − PRE)/PRE]. Change scores were significantly higher than zero for the “social positive” condition (median: 0.07, one sample Wilcoxon test, *p* = 0.010, *N* = 75) (Figure 1A, bottom), for “social negative” (median: 0.15, *p* = 0.006, *N* = 29) (Figure 1C, bottom), but were not significantly different from zero for the “non-social” condition (median: 0.03, *p* = 0.529, *N* = 46) (Figure 1B, bottom). Thus, the social aspect of the sensorimotor synchronization task, regardless of feedback type, is necessary for increasing OXT levels.

**FIGURE 2:**
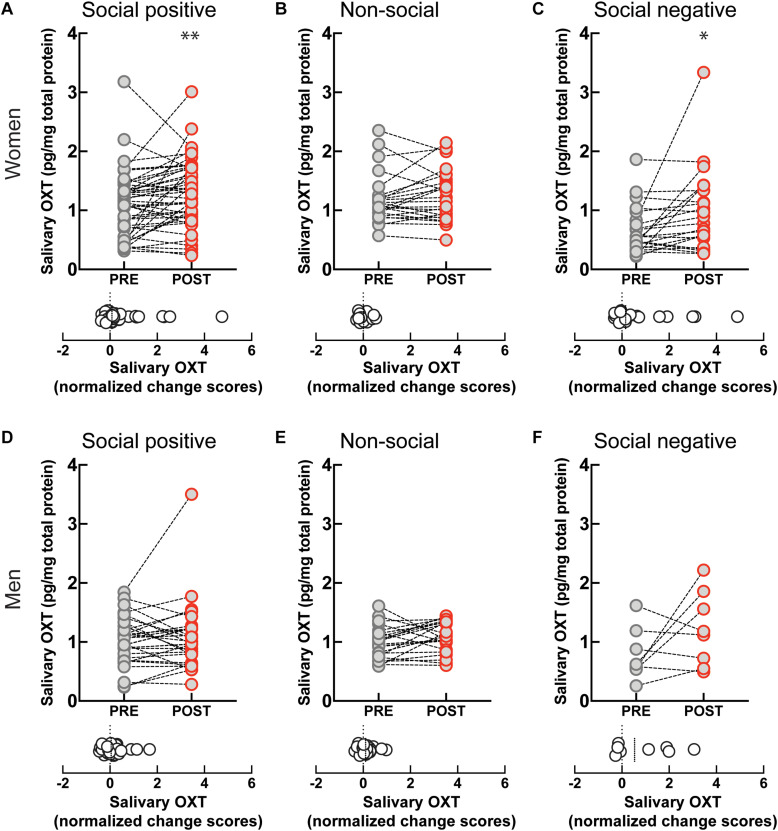
Gender-specific effect of sensoriomotor imitation on endogenous OXT concentrations: **(A)** women, “social positive” condition, **(B)** women, “non-social,” **(C)** women, “social negative,” **(D)** men, “social positive,” **(E)** men, “non-social,” and **(F)** men, “social negative.” OXT, oxytocin; PRE, before imitation; POST, after imitation. *p < 0.05, **p < 0.001.

To investigate if the OXT response to sensorimotor social synchronization is sexually dimorphic, we compared results in women and men. Following the “social positive” task, salivary OXT significantly increased in women (PRE: 1.09 ± 0.08 pg/mg, POST: 1.29 ± 0.08 pg/mg, *p* < 0.005, *N* = 45; median change score: 0.08, *p* = 0.012, *N* = 45) (Figure 2A), but not in men (PRE: 1.01 ± 0.07 pg/mg, POST: 1.06 ± 0.1 pg/mg, *p* = 0.626, *N* = 30; median change score: 0.05, *p* = 0.370, *N* = 30) (Figure 2D). Similarly, following the “social negative” task, salivary OXT significantly increased in women (PRE: 0.66 ± 0.08 pg/mg, POST: 0.98 ± 0.15 pg/mg, *p* = 0.019, *N* = 22; median change score: 0.15, *p* = 0.025, *N* = 22) (Figure 2C), and tended to also increase in men without reaching significance at the current sample size (PRE: 0.77 ± 0.15 pg/mg, POST: 1.21 ± 0.22 pg/mg, *p* = 0.382, *N* = 8; median change score: 0.53, *p* = 0.312, *N* = 8) (Figure 2F). Following the “non-social” task, OXT remained stable in both women (PRE: 1.19 ± 0.08 pg/mg, POST: 1.24 ± 0.08 pg/mg, *p* = 0.438, *N* = 24; median change score: 0.01, *p* = 0.302, *N* = 24) (Figure 2B), and men (PRE: 0.99 ± 0.05 pg/mg, POST: 1.08 ± 0.05 pg/mg, *p* = 0.112, *N* = 22; median change score: 0.1, *p* = 0.054, *N* = 22) (Figure 2E). This indicates that in women, the oxytocinergic system is more receptive to social sensorimotor synchronization.

Artifactual differences might appear between conditions due to batch processing of saliva samples, and due to variability in un-probed dimensions of the “social positive” vs “social negative” sample and the “social positive” vs “non-social” sample. To eliminate this possibility, we performed a within subject paired analysis of change scores (Supplementary Figure S4). We found that “social positive” and “social negative” conditions similarly increased change scores in women (“social positive” median change score: 0.1, “social negative” median change score: 0.15, Wilcoxon matched-pairs signed rank test, *p* = 0.785, *N* = 21) (Supplementary Figure S4A), and acted similarly in men (“social positive” median change score: 0.42, “social negative” median change score: 0.53, *p* = 0.312, *N* = 8) (Supplementary Figure S4B). On the contrary, “social positive” condition led to significantly higher change scores than the “non-social” in women (“social positive” median change score: 0.07, “non-social” median change score: −0.01, *p* = 0.005, *N* = 24) (Supplementary Figure S4C), but not in men (“social positive” median change score: 0.03, “non-social” median change score: 0.1, *p* = 0.222, *N* = 22) (Supplementary Figure S4D). Similar results were obtained using a repeated measure two-way ANOVA analysis (Supplementary Figure S5). For women, there was a significant effect of time (PRE vs POST) in the “social positive” vs “social negative” sample [*F*(1, 20) = 13.01, *p* = 0.001; Holm-Sidak test for “social positive,” *p* = 0.074, and for “social negative,” *p* = 0.035] (Supplementary Figure S5A), and also in the “social positive” vs “non-social” sample [*F*(1, 23) = 5.709, *p* = 0.025; Holm-Sidak test for “social positive,” *p* = 0.048, and for “non-social,” *p* = 0.388] (Supplementary Figure S5C). For men, two-way ANOVA analysis did not show clearly significant results between PRE and POST salivary OXT level, in either the “social positive” vs “social negative” sample [*F*(1, 7) = 5.564, *p* = 0.051] (Supplementary Figure S5B), or in the “social positive” vs “non-social” sample [*F*(1, 21) = 0.029, *p* = 0.865] (Supplementary Figure S5D).

### Dissociated Effects of Sensorimotor Synchronization on Closeness and on Salivary OXT

**FIGURE 3:**
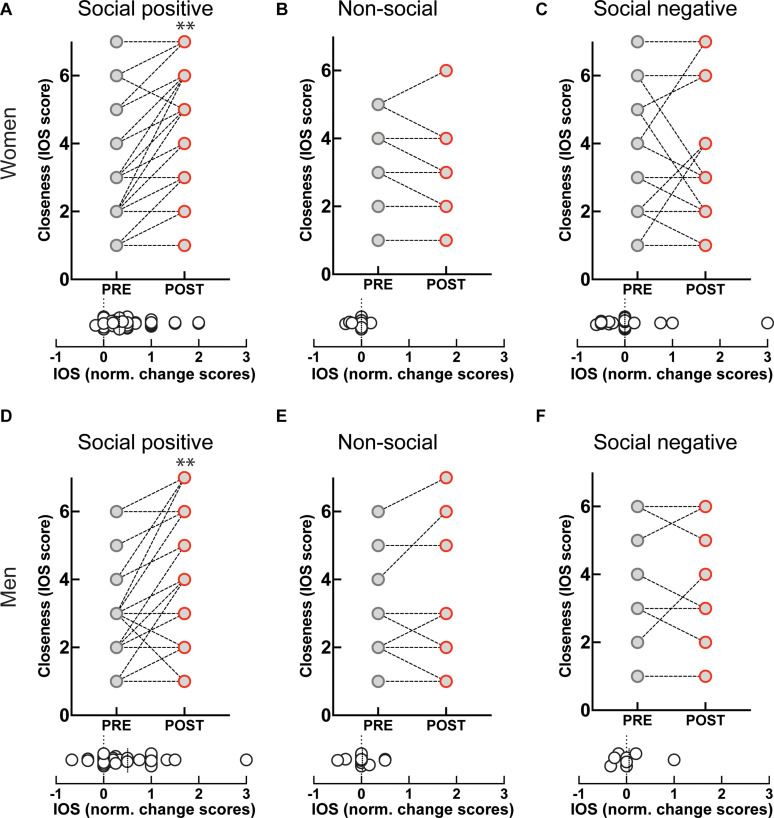
Effect of sensoriomotor imitation on closeness ratings by gender: **(A)** women, “social positive” condition, **(B)** women, “non-social,” **(C)** women, “social negative,” **(D)** men, “social positive,” **(E)** men, “non-social,” **(F)** men, “social negative.” IOS, inclusion of other in the self-scale**; Pre, before imitation; Post, after imitation. **p < 0.001.

Engagement in the “social positive” task prompted a large increase in perceived closeness to the interaction partner for both women (PRE: 2.79 ± 0.2, POST: 3.85 ± 0.2, *p* < 0.0001, *N* = 49; median change score: 0.33, *p* < 0.0001, *N* = 49) (Figure 3A), and men (PRE: 2.9 ± 0.2, POST: 3.8 ± 0.3, *p* = 0.0005, *N* = 31; median change score: 0.25, *p* = 0.0002, *N* = 31) (Figure 3D). The “non-social” condition registered no change in closeness to a person they have not interacted with in both women (PRE: 2.3 ± 0.3, POST: 2.2 ± 0.3, *p* = 0.625, *N* = 19; median change score: 0, *p* = 0.375, *N* = 19) (Figure 3B), and men (PRE: 2.5 ± 0.3, POST: 2.6 ± 0.4, *p* = 0.750, *N* = 16; median change score: 0, *p* = 0.812, *N* = 16) (Figure 3E). This shows that engagement in a sensorimotor non-social synchronization task does not lead to non-specific effects on social closeness.

Unexpectedly, although imitation with negative feedback leads to a significant increase in salivary OXT, it does not change closeness scores either in women (PRE: 3.04 ± 0.37, POST: 2.95 ± 0.40, *p* = 0.826, *N* = 22; median change score: 0, *p* = 0.881, *N* = 22) (Figure 3C), or men (PRE: 3.75 ± 0.64, POST: 3.75 ± 0.64, *p* > 0.999, *N* = 8; median change score: 0, *p* > 0.999, *N* = 8) (Figure 3F). Furthermore, when we fitted linear regression models with OXT dynamic (change) scores as dependent variable and pre-intervention measure as a covariate for both women and men, we found no significant link between them (β = 0.03, *t*(72) = 1.1, *p* = 0.286). This indicates that the increase in endogenous OXT following the “social positive” task cannot be fully explained by the increase in perceived closeness to the interaction partner.

In order to investigate other aspects of social behavior, on a subgroup of subjects we assessed generosity using the DG task (Brocklebank et al., 2011). Participants played marginally more egoistically after negative feedback (PRE: 1.67 ± 0.45, POST: 1.03 ± 0.27, *p* = 0.055, *N* = 30), while positive feedback had no effect (PRE: 1.82 ± 0.43, POST: 1.53 ± 0.42, *p* = 0.474, *N* = 30). Nevertheless, in the positive feedback condition, change scores in OXT levels correlated with those on DG [*r*(27) = −0.44, *p* = 0.017].

### Effects of Social Imitation on Stress and Well-Being

In both humans and animal models, OXT was shown to have an anxiolytic effect (Viviani et al., 2011; Cardoso et al., 2012, 2013). Based on these and on our biochemical findings, we would expect that perceived momentary stress decreases after social sensorimotor synchronization with either positive or negative feedback, but not after non-social synchronization. Similarly, we would expect perceived momentary well-being to increase following social synchronization but not following “non-social” task.

**FIGURE 4:**
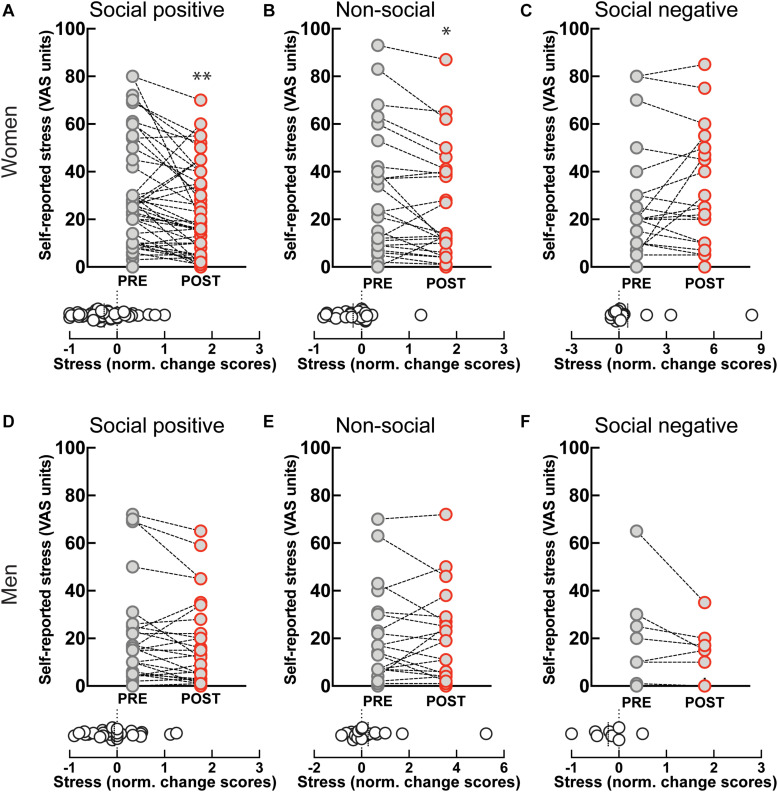
Effect of sensoriomotor imitation on momentary stress levels by gender: **(A)** women, “social positive” condition, **(B)** women, “non-social,” **(C)** women, “social negative,” **(D)** men, “social positive,” **(E)** men, “non-social,” **(F)** men, “social negative.” VAS, visual analog scale; Pre, before imitation; Post, after imitation. *p < 0.05, **p < 0.001.

Self-reported stress levels showed a significant decrease after “social positive” condition in women (PRE: 28.61 ± 3.2, POST: 21.39 ± 2.67, *p* = 0.0001; *N* = 49; median change scores: −0.2, *p* = 0.001, *N* = 49) (Figure 4A), but not in men (PRE: 18.16 ± 3.72, POST: 15.77 ± 3.24, *p* = 0.058, *N* = 31; median change scores: 0, *p* = 0.372, *N* = 31) (Figure 4D). However, perceived stress also decreased after the “non-social” condition in women (PRE: 29.46 ± 5.21, POST: 24.54 ± 4.57, *p* = 0.017, *N* = 26; median change score: −0.21, *p* = 0.009, *N* = 26) (Figure 4B), and not in men (PRE: 18.78 ± 4.27, POST: 17.43 ± 3.92, *p* = 0.8734, *N* = 23; median change score: 0, *p* = 0.798, *N* = 23) (Figure 4E). Although the change after the “social positive” condition had stronger significance, an ANCOVA test with pre-intervention measure entered as a covariate to control for individual variability did not establish a significant difference between stress changes in the two experimental conditions [*F*(1, 95) = 0.79, *p* = 0.376, $\eta_{p}^{2}$ = 0.01]. These results show that behavioral engagement in either social or non-social sensorimotor synchronization tasks can decrease stress, in dissociation with changes in salivary OXT. The observed decrease in stress is unlikely to simply be a product of passing time, independent of task, as perceived stress does not decrease after “social positive” task in either women (PRE: 25.55 ± 5.18, POST: 29.14 ± 5.28, *p* = 0.630, *N* = 22; median change score: 0, *p* = 0.810, *N* = 22) (Figure 4C), or men (PRE: 20.13 ± 7.45, POST: 14 ± 4, *p* = 0.187, *N* = 8; median change scores: −0.17, *p* = 0.250, *N* = 8) (Figure 4F).

**FIGURE 5:**
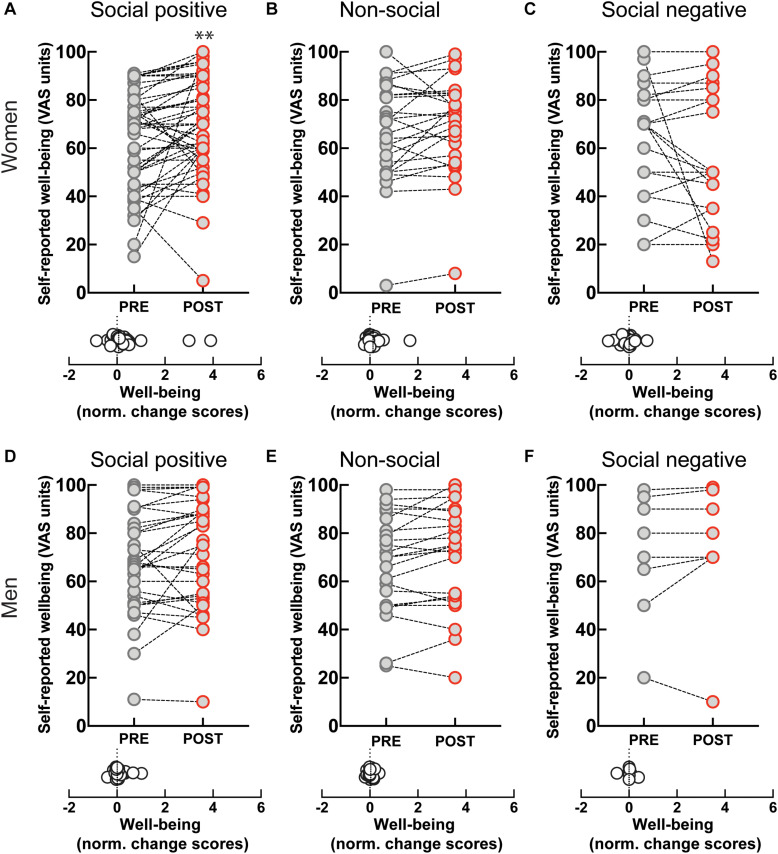
Effect of sensoriomotor imitation on momentary well-being ratings by gender: **(A)** women, “social positive” condition, **(B)** women, “non-social,” **(C)** women, “social negative,” **(D)** men, “social positive,” **(E)** men, “non-social,” **(F)** men, “social negative.” VAS, visual analog scale; Pre, before imitation; Post, after imitation. **p < 0.001.

Perceived well-being increased after the ‘social positive’ condition in women (PRE: 62.88 ± 2.9, POST: 69.33 ± 2.87, *p* < 0.003, *N* = 49; median change score: 0.06, *p* = 0.003, *N* = 49) (Figure 5A), but not in men (PRE: 67.68 ± 3.88, POST: 71.19 ± 4.02, *p* = 0.093, *N* = 31; median change score: 0.01, *p* = 0.148, *N* = 31) (Figure 5D). On the contrary, perceived well-being did not robustly change after the control “non-social” condition either in women (PRE: 66.62 ± 4.1, POST: 70 ± 3.87, *p* = 0.065, *N* = 26; median change score: 0.03, *p* = 0.038, *N* = 26) (Figure 5B), or in men (PRE: 67.87 ± 4.27, POST: 70.35 ± 4.53, *p* = 0.111, *N* = 23; median change score: 0.01, *p* = 0.165, *N* = 23) (Figure 5E). These data suggest that the increase in well-being might correlate with increases in endogenous OXT. Indeed, in women experiencing social synchronization with positive feedback we find a positive correlation between well-being and OXT change scores [*r*(45) = 0.33, *p* = 0.025].

Despite the increase in endogenous OXT experienced after “social negative” condition, we found no significant change in perceived well-being either in women (PRE: 62.14 ± 5.07, POST: 56 ± 5.6, *p* = 0.551, *N* = 22; median change score: 0, *p* = 0.440, *N* = 22) (Figure 5C), or in men (PRE: 71 ± 9.28, POST: 73.38 ± 10.03, *p* = 0.437, *N* = 8; median change score: 0, *p* = 0.625, *N* = 8) (Figure 5F). Taken together, our data indicate that endogenous OXT is not sufficient in predicting behavioral outcomes from the social synchronization task, but that in the context of positive feedback it can predict increases in measured generosity and perceived well-being.

### Personality Factors Affect Behavioral and Biochemical Outcomes

We also explored if changes in outcome measures could be accounted for by personality traits or depressive and anxious symptoms. Thus, we fitted linear regression models with dynamic (change) scores as dependent variable and pre-intervention measure as a covariate. We found that trait anxiety is predictive of increased stress after the “social positive” condition [β = 0.26, *t*(47) = 2.13, *p* = 0.038], but not after the “non-social” condition [β = −0.14, *t*(46) = −0.94, *p* = 0.348]. Similarly, higher depression scores predicted lower well-being benefits after the “social positive” condition [β = −0.62, *t*(47) = −2.38, *p* = 0.022], while remaining uninformative for the “non-social” condition [β = −0.11, *t*(46) = −0.54, *p* = 0.594]. This led us to conclude that even simple social interaction tasks, while generally beneficial, may elicit uncomfortable role demands in people with certain traits or dispositions. Further studies should pay careful attention to these covariates.

For the “social negative” condition, *Neuroticism* predicted an increase in stress [β = 0.20, *t*(26) = 2.68, *p* = 0.012], while *Conscientiousness* predicted a decrease [β = −0.21, *t*(26) = −2.56, *p* = 0.016]. More specifically, the *Competence* [β = −1.03, *t*(26) = −2.36, *p* = 0.026] and *Order* [β = −0.90, *t*(26) = −2.34, *p* = 0.027] traits from the domain of *Conscientiousness* seemed to be potent predictors. Similarly, in the “social negative” condition, both *Angry Hostility* [β = −1.23, *t*(26) = −2.13, *p* = 0.043] and *Gregariousness* [β = −1.65, *t*(26) = −2.46, *p* = 0.021] predicted a decrease in well-being, while *Modesty* [β = 0.09, *t*(26) = −2.14, *p* = 0.042] predicted a modest increase in perceived closeness. The apparent lack of change in behavioral measures after the “social negative” task appear to result from heterogeneous effects contingent on personality traits. For certain personalities, imitation with negative feedback can prove to be behaviorally beneficial.

For the “social positive” condition, we fitted linear regression models with OXT dynamic (change) scores as dependent variable and pre-intervention personality measure as a covariate for both women and men. Personality traits weakly predicted OXT change. In women, OXT change was predicted by *Assertiveness* [β = −0.03, *t*(38) = −2.04, *p* = 0.048], *Dutifulness* [β = −0.04, *t*(38) = −2.58, *p* = 0.013], and *Self-Discipline* [β = −0.04, *t*(38) = −2.61, *p* = 0.012]. In men, OXT change was only predicted by *Impulsiveness* [β = −0.03, *t*(22) = −2.53, *p* = 0.019]. For the whole sample, both genders, only *Assertiveness* [β = −0.03, *t*(63) = −2.56, *p* = 0.012] significantly predicted OXT change.

### Interactions Between Behavioral Changes Following Social Sensorimotor Synchronization

We found that change scores in stress and closeness, as well as well-being and closeness were significantly correlated for women (*N* = 49), but not for men (*N* = 31) (see Table 1). The failure to reach statistical significance could be accounted by the fact that the male sample was smaller. Nevertheless, the pattern of correlation could be indicative of different modulation mechanisms for men and women especially concerning the relationship between stress and OXT on one hand, and stress and closeness on the other.

**TABLE 1:** Correlations between change scores due to imitation among women and men.

**Variable**	**Gender**	**ΔOXT**	**ΔStress**	**ΔWell-being**	**ΔCloseness**
ΔStress	F	0.02			
	M	−0.20			
ΔWell-being	F	0.01	−0.30*		
	M	0.11	−0.20		
ΔCloseness	F	0.02	−0.33*	0.23	
	M	−0.13	−0.16	−0.01	

*p < 0.05.

Given that OXT was not significantly linked to stress and closeness variables we proceeded to test if the temporal changes in stress were mediated by shifts in perceived closeness following the social synchronization task. To examine indirect effects of time (i.e., pre/post task) changes in stress via modifications in closeness, we applied the recently developed within participant mediation framework developed by Yzerbyt et al. (2018). Inferences about within-subject mediation are based on a series of hypothesis tests (i.e., component approach; Judd et al., 2001) as to not inflate type I errors (Yzerbyt et al., 2018). Accordingly, first it is required to establish the presence of an indirect effect by means of the significance of both individual components (i.e., the joint-significance test concerning paths *a* and *b*), and only after proceed with Monte Carlo resampling to compute the confidence interval for the indirect effect (i.e., the product of the two estimated components – *ab*). This analysis was conducted using the *JSmediation* package for R (Yzerbyt et al., 2018).

**FIGURE 6:**
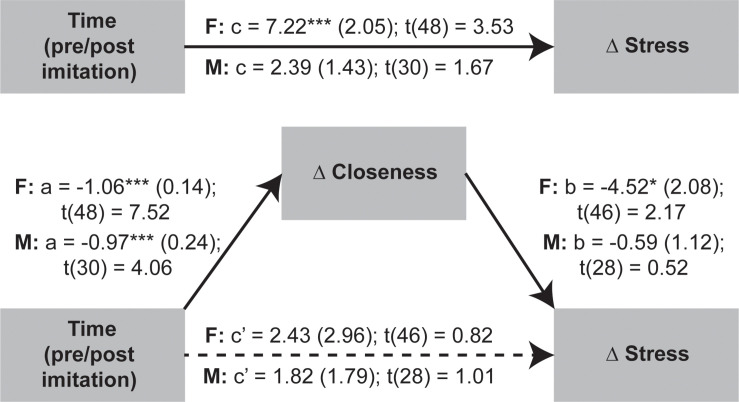
Investigation of imitation effects on stress changes mediated by modifications in perceived closeness using within-participant mediation model proposed by Yzerbyt et al. (2018). The SEs of point estimates are provided in parentheses. *p < 0.05, ***p < 0.001.

The applied within-participant mediation model confirmed that observed increases in closeness fully accounted for the decreases in stress, but only for females (Figure 6). The within-participant indirect effect for women was estimated by Monte Carlo method and was found to be significant (*ab* = 4.8; 95% CI = [0.56; 9.74]), while the same effect was non-significant for men.

## Discussion

The major aim of the current research was to develop further a behavioral intervention that could lead to the release of endogenous OXT and that could be used as therapy for afflictions with a social and/or stress component. We built on previous findings showing the beneficial effects of social sensorimotor synchronization tasks (Aoki and Yamasue, 2015; Hale and Hamilton, 2016; Spengler et al., 2017). However, unlike most previous studies, we focused on responses triggered in *imitator* rather than in *imitee*.

We find that positive feedback leads to increased salivary OXT, increased well-being, and increased closeness to the interaction partner. None of these results were reproduced when subjects had to synchronize their movements with a geometrical figure displayed on a computer screen, a non-social control. However, negative social feedback can also increase salivary OXT, although to a lower degree, indicating that, in fact, social approval during interactions is not needed for increasing salivary OXT. This finding is consistent with previous reports that synchronization without any type of feedback can increase salivary OXT in both *imitee* and *imitator* (Spengler et al., 2017), suggesting that perhaps a perception-action matching mechanism involving the mirror neuron system (Decety et al., 2002; Rizzolatti and Craighero, 2004; Molenberghs et al., 2009; Hogeveen et al., 2014; Hale and Hamilton, 2016) might be involved in triggering hormonal responses.

Despite the increase in endogenous OXT following both types of social synchronization tasks, behavioral changes were heavily linked to the type of feedback subjects received. Thus, well-being and closeness increased, while stress decreased after synchronization with social approval, but not after synchronization with social disapproval. Similarly, although social approval did not change prosocial behaviors (altruism and generosity as measured with the DG), social disapproval made subjects less altruistic and more egotistical. These findings indicate that behavioral responses to sensorimotor synchronization depend on the perceived “value” of the interaction, supporting a reward-based mechanism (Hale and Hamilton, 2016).

Another important result of our study is that both hormonal and behavioral changes induced by social sensorimotor synchronization are sexually dimorphic. This supports previous findings showing highly complex actions of OXT with regard to sex differences in behavior (Caldwell, 2018). OXT levels and OXT release tend to be higher in females (Kramer et al., 2004; Carter, 2007; Marazziti et al., 2019). However, regulation of systems that rely on OXT seem to be sexually dimorphic in some species, but not all (Kramer et al., 2004), while sex differences in behavior are seldomly associated with structural changes in the OXT system (Caldwell, 2018). Anxiolytic effects of OXT have been found both in women and in men (Ditzen et al., 2009; Cardoso et al., 2012; Kubzansky et al., 2012), while some research suggests that OXT mediates behavioral responses to stress more strongly in women than in men, which may result in female-specific tend-and-befriend coping strategies (Taylor et al., 2000; Cardoso et al., 2013). More so, studies point toward other interesting sex-specific patterns of behavior linked to OXT (Preckel et al., 2014; Rilling et al., 2014; Scheele et al., 2014). For example, Morhenn et al. (2008) found that women were more susceptible than men to OXT release and monetary sacrifice during DG after physical touch.

In addition to gender differences, we also find that personality traits could play a crucial role in both behavioral and hormonal responses. Perhaps most strikingly, we find that social disapproval is beneficial for certain personalities, indicating that perhaps some individuals interpret the negative social feedback as being instructive rather than punitive. Although these findings remain exploratory, they generate testable hypotheses for future, larger-scale studies.

Our results indicate that distinct neurocognitive mechanisms might account for hormonal changes and for behavioral changes induced by social sensorimotor synchronization. Moreover, we find crucial gender and personality type differences underlying these changes. Our results could provide crucial information on optimizing and individualizing behavioral therapies for afflictions of social interaction and stress management.

### Study Limitations

The present study has several limitations. The small sample size provided enough statistical power to detect significant differences in the imitation conditions, but not enough to confidently test differences in gains across conditions. Another limitation concerns the findings regarding the link between personality traits and our intervention outcome variables. Stemming from an exploratory analysis involving multiple testing, the risk of type-1 errors is high and the results should be interpreted with caution. However, these findings could generate future research, a contribution to the field that would otherwise be negated by restrictive corrections that amplifies type-2 error rates.

Although not suggested by more in depth analysis of our current sample, crossover effects are a threat in within-participant designs. Most notably, comparisons between a positive-feedback interaction and a negative-feedback interaction may be subjected to referencing effects, regardless of efforts to randomize and counterbalance the condition order. In our present study the only precaution against such an effect was temporal spacing of tests. Future studies of social interaction with modulated feedback should use designs able to adequately test for crossover effects.

Salivary OXT may not reflect changes in brain levels of the hormone (Neumann and Landgraf, 2012; Neumann and Slattery, 2016; Schladt et al., 2017), as neuronal populations releasing the hormone in the periphery are different from those releasing it centrally. Moreover, OXT does not cross the blood brain barrier (Leng and Ludwig, 2016). Measuring OXT release in the cerebrospinal liquid is invasive and not ethical in healthy individuals. Given this, in pilot experiments we compared measurements of OXT in saliva, blood and urine. We, as others before us (Holt-Lunstad et al., 2008; White-Traut et al., 2009; Grewen et al., 2010; Feldman et al., 2011; Hoffman et al., 2012), found similar levels and ranges of OXT between these internal fluids (data not shown). We chose to sample saliva to ensure comfortable conditions for our subjects, and thus to increase retention in the study.

## Data Availability Statement

The datasets generated for this study are available on request to the corresponding author.

## Ethics Statement

The studies involving human participants were reviewed and approved by Ethics Committee of the University of Theatre and Film “I. L. Caragiale” Bucharest. The patients/participants provided their written informed consent to participate in this study.

## Author Contributions

All authors contributed to the design of the experiment and interpretation of results. CP (1st author) analyzed all behavioral data. CP (4th author) analyzed biochemical data. AS, RB, MT, and CP (6th author) conducted the experiments and data collection. CP (1st author), IP, and IC wrote the manuscript, with feedback from all authors. All authors contributed to the article and approved the submitted version.

## Conflict of Interest

The authors declare that the research was conducted in the absence of any commercial or financial relationships that could be construed as a potential conflict of interest.

## Supplementary Material

FIGURE S1 Age and gender distribution in the recruited sample.

FIGURE S2 Diagram of the experimental procedure. Subjects participated to the experiment on two different days set at least 1 week apart and at most 10 days apart. Self-report measure completion (stress VAS, well-being VAS, IOS) and saliva sampling followed the same timeline for both days. Personality assessment was done only at the beginning of the first experimental day. Participants were randomized to treatment (“social positive imitation”) or control conditions (“social negative imitation”/“non-social imitation”), as well as to the order they would experience these conditions (day_1_/day_2_). The Dictator Game task was administered only to participants allocated to the group that would be used to investigate social imitation with modulated feedback.

FIGURE S3 Distribution of personality traits in the recruited sample. Personality traits are listed on the *x*-axis. *T* scores (M=50, SD=10) are displayed on *y*-axis. Shaded areas are indicative of intensity categories for the Romanian normative sample (both genders): 20–35 *very low* scores, 35–45 *low* scores, 45–55 *average*, 55–65 *high* scores, and 65–80 *very high* scores. N, neuroticism; E, extraversion; O, openness to experience; A, agreeableness; C, conscientiousness; N1, anxiety; N2, hostility/anger; N3, depression; N4, self-consciousness; N5, impulsiveness; N6, vulnerability to stress; E1, warmth; E2, gregariousness; E3, assertiveness; E4, activity; E5, excitement seeking; E6, positive emotion; O1, fantasy; O2, aesthetics; O3, feelings; O4, actions; O5, ideas; O6, values; A1, trust; A2, straightforwardness; A3, altruism; A4, compliance; A5, modesty; A6, tendermindedness; C1, competence; C2, order; C3, dutifulness; C4, achievement striving; C5, self-discipline; C6, deliberation.

FIGURE S4 Differences in normalized change scores between conditions. **(A)** Women, “social positive” vs “social negative,” **(B)** men, “social positive” vs “social negative,” **(C)** women, “social positive” vs “non-social,” **(D)** men, “social positive” vs “non-social.”

FIGURE S5 Repeated measure two-way ANOVA analysis for normalized (to daily values) salivary oxytocin. **(A)** Women, “social positive” vs “social negative,” **(B)** men, “social positive” vs “social negative,” **(C)** women, “social positive” vs “non-social,” **(D)** men, “social positive” vs “non-social.”

TABLE S1 Summary of pre-post comparisons within experimental conditions (Wilcoxon matched-pairs signed rank test).
