# Supplementary material for: Social Feedback During Sensorimotor Synchronization Changes Salivary Oxytocin and Behavioral States
Source: Front Psychol. 2020 Sep 23;11:531046. doi: 10.3389/fpsyg.2020.531046 (PMC7538614; doi:10.3389/fpsyg.2020.531046)
Supplement: FIGURE S1 — Age and gender distribution in the recruited sample. [file Data_Sheet_1.PDF]

Social Feedback during Sensorimotor Synchronization Changes Salivary Oxytocin and Behavioral States

Supplementary material:

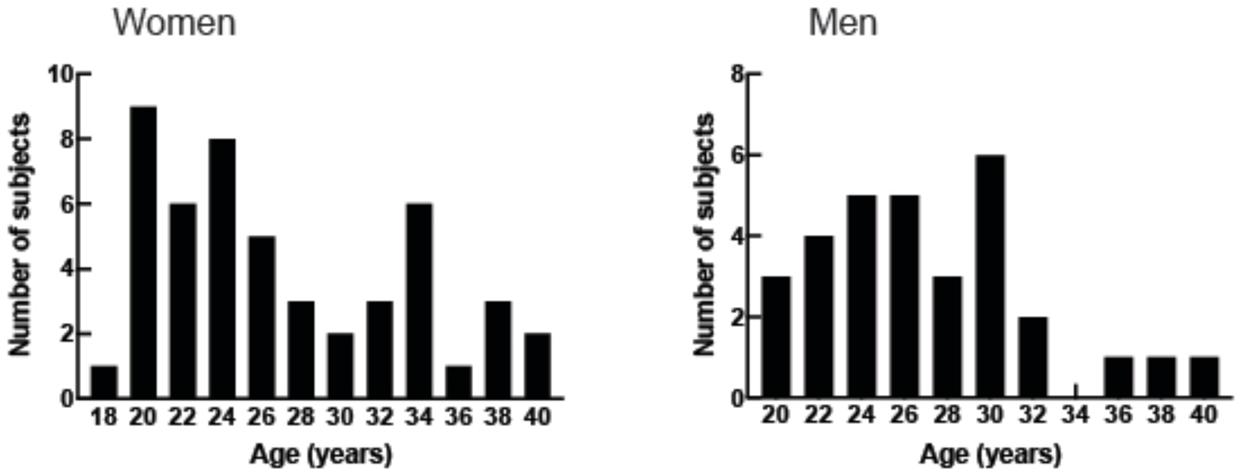

Figure S1

Figure S1: Age and gender distribution in the recruited sample.

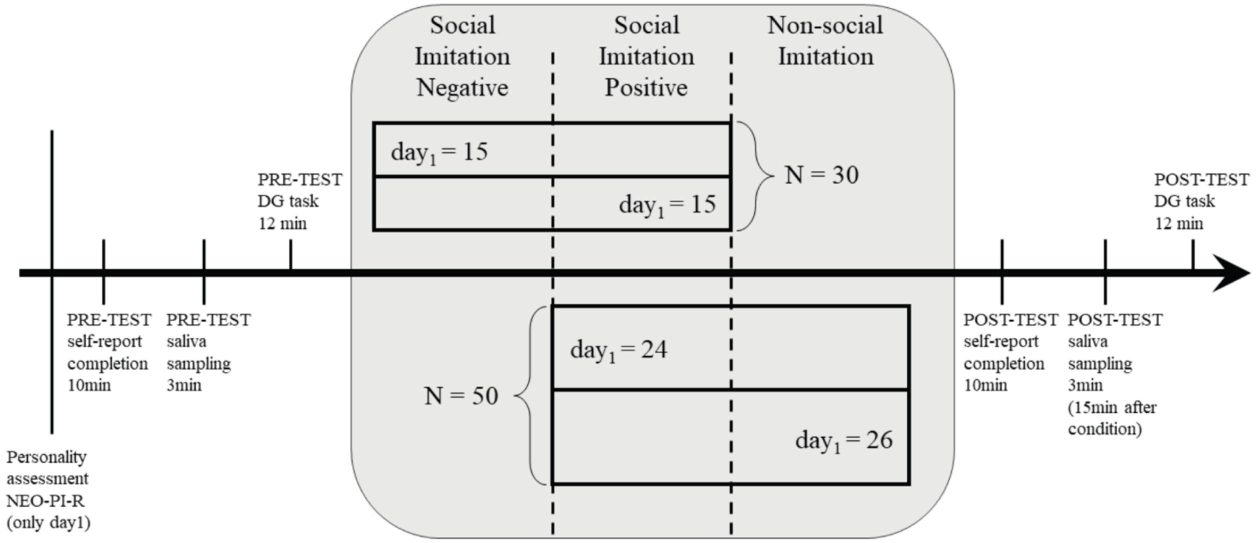

Figure S2: Diagram of the experimental procedure. Subjects participated to the experiment on two different days set at least one week apart and at most 10 days apart. Self-report measure completion (stress VAS, well-being VAS, IOS) and saliva sampling followed the same timeline for both days. Personality assessment was done only at the beginning of the first experimental day. Participants were randomized to treatment ('social positive imitation') or control conditions ('social negative imitation' / 'non-social imitation'), as well as to the order they would experience these conditions (day_1/day_2). The Dictator Game task was administered only to participants allocated to the group that would be used to investigate social imitation with modulated feedback.

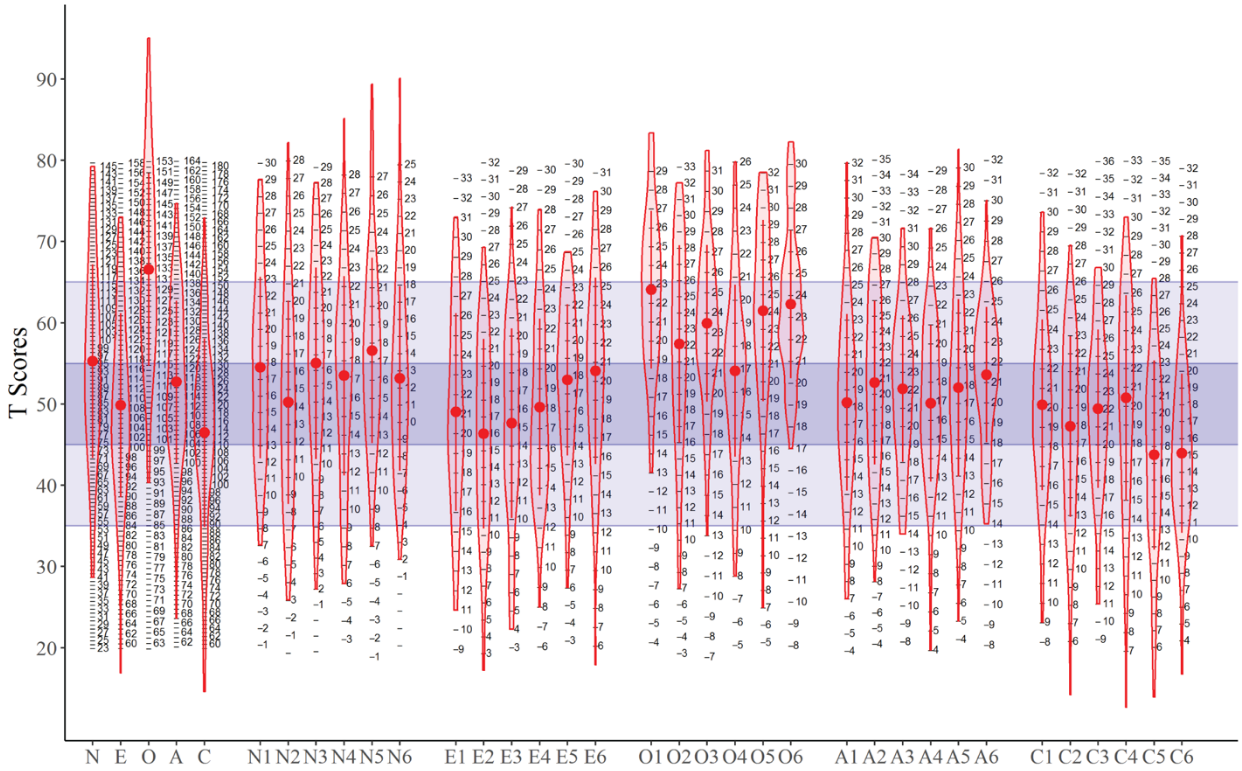

Figure S3: Distribution of personality traits in the recruited sample. Personality traits are listed on the x-axis. T scores (M=50, SD=10) are displayed on y-axis. Shaded areas are indicative of intensity categories for the Romanian normative sample (both genders): 20-35 *very low* scores, 35-45 *low* scores, 45-55 *average*, 55-65 *high* scores, and 65-80 *very high* scores.

N=Neuroticism, E=Extraversion, O=Openness to experience, A=Agreeableness, C=Conscientiousness; N1=Anxiety, N2=Hostility/Anger, N3=Depression, N4=Self-consciousness, N5=Impulsiveness, N6=Vulnerability to Stress; E1=Warmth, E2=Gregariousness, E3=Assertiveness, E4=Activity, E5=Excitement Seeking, E6=Positive Emotion; O1= Fantasy, O2=Aesthetics, O3=Feelings, O4=Actions, O5=Ideas, O6=Values; A1=Trust, A2=Straightforwardness, A3=Altruism, A4=Compliance, A5=Modesty, A6=Tendermindedness; C1=Competence, C2=Order, C3=Dutifulness, C4=Achievement Striving, C5=Self-Discipline, C6=Deliberation.

Table S1. Summary of Pre-Post comparisons within experimental conditions (Wilcoxon matched-pairs signed rank test).

		Social positive				Non-social				Social negative			
		N	Pre	Post	p	N	Pre	Post	p	N	Pre	Post	p
**OXT**	F	45	1.09 (0.56)	1.28 (0.57)	0.006 **	24	1.19 (0.42)	1.24 (0.43)	0.439 ns	22	0.66 (0.40)	0.98 (0.70)	0.019 *
	M	30	1.01 (0.40)	1.06 (0.57)	0.626 ns	22	0.99 (0.27)	1.08 (0.26)	0.113 ns	8	0.77 (0.43)	1.21 (0.62)	0.383 ns
	∑	75	1.06 (0.50)	1.19 (0.58)	0.009 **	46	1.09 (0.37)	1.17 (0.36)	0.082 ns	30	0.69 (0.41)	1.04 (0.68)	0.016 *
**Cl**	F	49	2.79 (1.45)	3.85 (1.68)	<0.001 ***	19	2.31 (1.37)	2.21 (1.35)	0.424 ns	22	3.04 (1.75)	2.95 (1.91)	0.785 ns
	M	31	2.90 (1.66)	3.87 (1.96)	<0.001 ***	16	2.56 (1.45)	2.68 (1.85)	0.572 ns	8	3.75 (1.83)	3.75 (1.83)	>0.999 ns
	∑	80	2.83 (1.53)	3.86 (1.78)	<0.001 ***	35	2.42 (1.39)	2.42 (1.59)	>0.999 ns	30	3.23 (1.77)	3.16 (1.89)	0.810 ns
**St**	F	49	28.61 (22.46)	21.38 (18.71)	<0.001 ***	26	29.46 (26.59)	24.53 (23.31)	0.019 *	22	25.54 (24.31)	29.13 (24.77)	0.631 ns
	M	31	18.16 (20.72)	15.77 (18.04)	0.060 ns	23	18.17 (19.94)	18.13 (19.18)	0.879 ns	8	20.12 (21.08)	14 (11.31)	0.172 ns
	∑	80	24.56 (22.26)	19.21 (18.54)	<0.001 ***	49	24.16 (24.15)	21.53 (21.49)	0.051 ns	30	24.10 (23.26)	25.10 (22.84)	0.782 ns
**WB**	F	49	62.87 (20.322)	69.32 (20.13)	0.005 **	26	66.61 (20.93)	70 (19.76)	0.067 ns	22	62.13 (23.80)	56 (26.30)	0.550 ns
	M	31	67.67 (21.63)	71.19 (22.39)	0.094 ns	23	67.87 (20.48)	70.34 (21.76)	0.112 ns	8	71 (26.25)	73.37 (28.37)	0.418 ns
	∑	80	64.73 (20.83)	70.05 (20.91)	<0.001 ***	49	67.20 (20.51)	70.16 (20.50)	0.017 *	30	64.5 (24.34)	60.63 (27.50)	0.793 ns

* p < 0.05, ** p < 0.01, *** p < 0.001, *ns* not statistically significant
F = Females, M = Males, ∑ = total sample (females and males)
Mean (SD)
OXT = Oxytocin, Cl = Closeness, St = Stress, WB = Well-Being

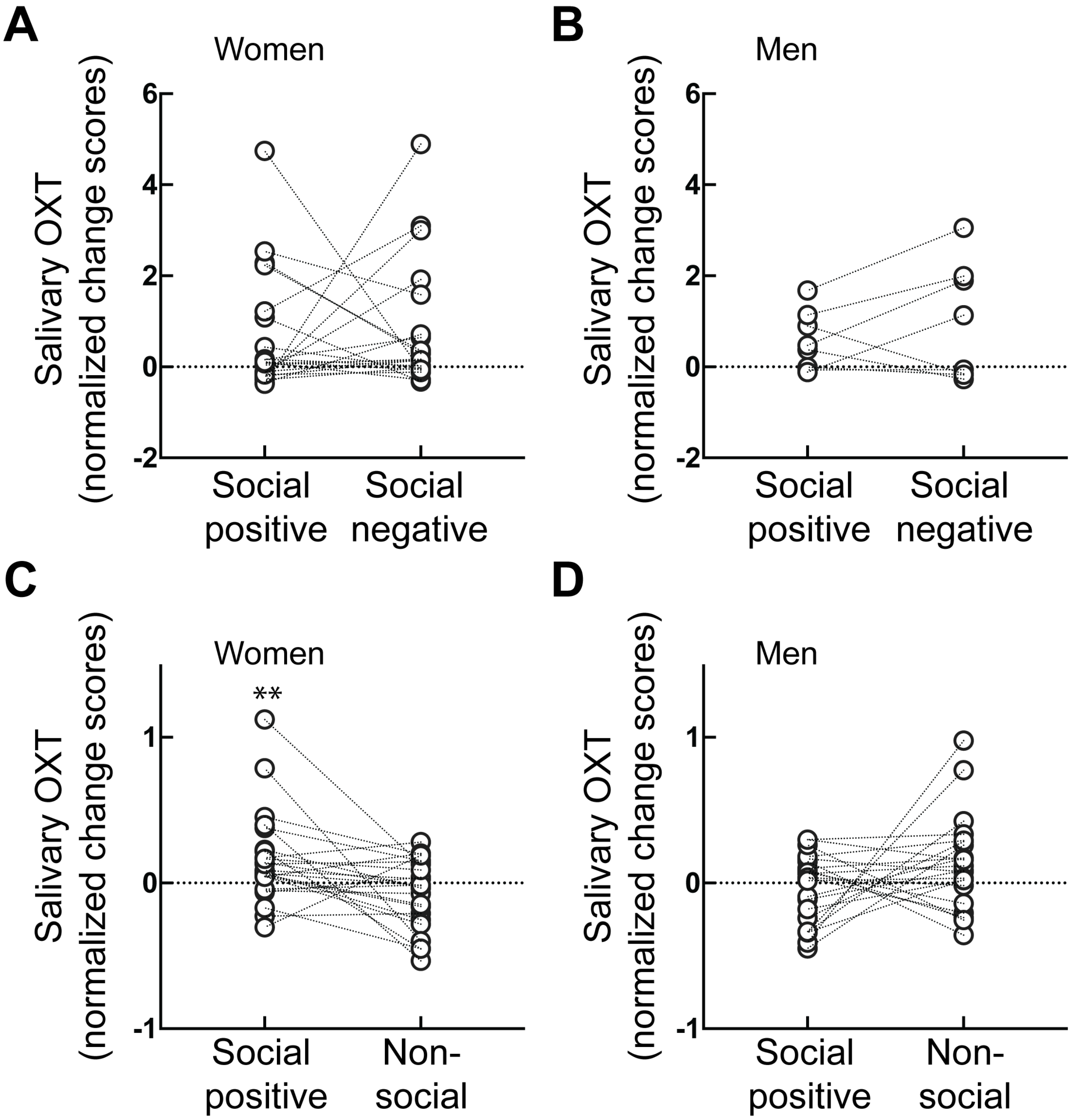

Figure S4: Differences in normalized change scores between conditions. (A) Women, 'social positive' vs 'social negative', (B) Men, 'social positive' vs 'social negative', (C) Women, 'social positive' vs 'non-social', (D) Men, 'social positive' vs 'non-social'.

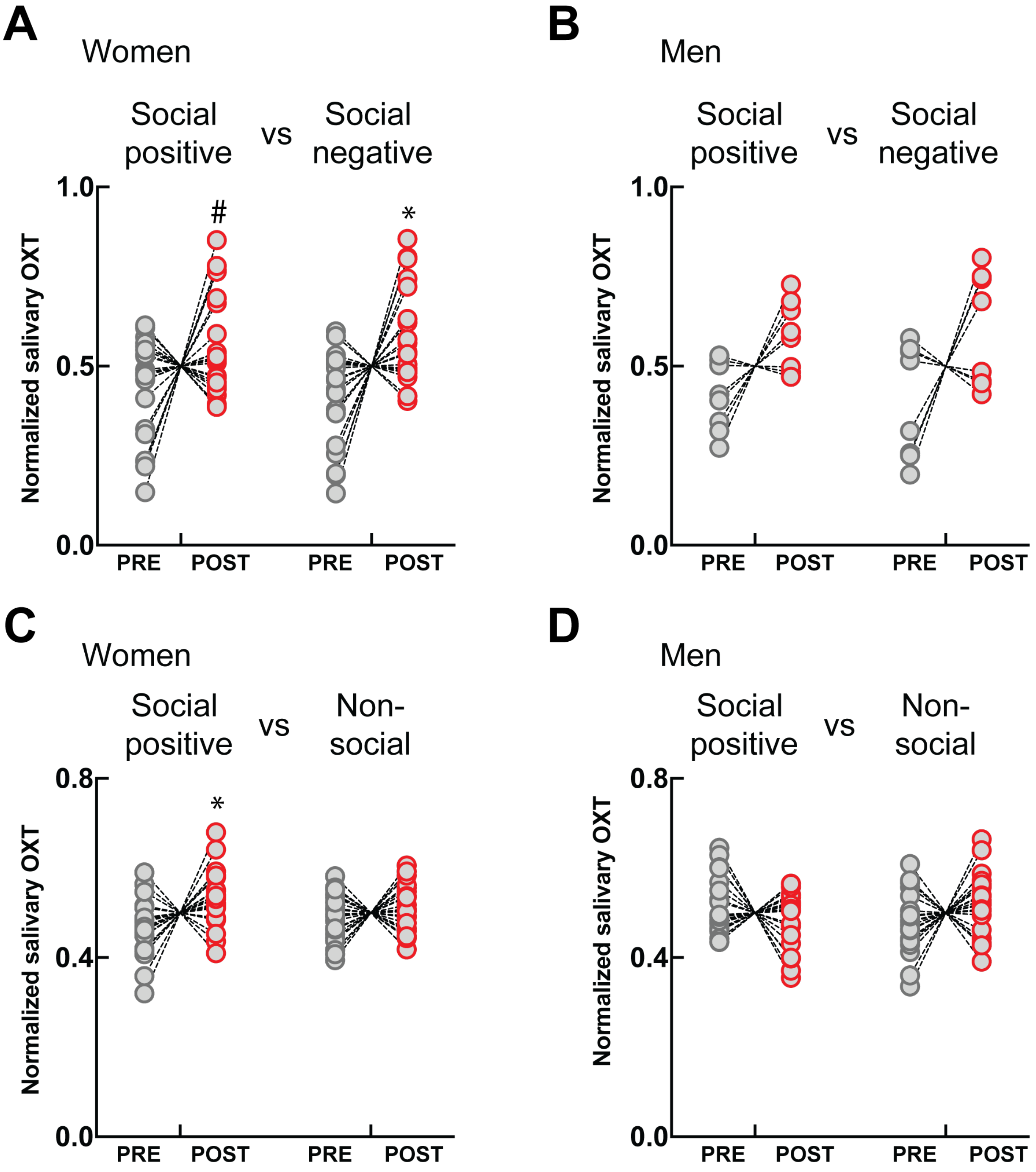

Figure S5: Repeated measure two-way ANOVA analysis for normalized (to daily values) salivary oxytocin. (A) Women, 'social positive' vs 'social negative', (B) Men, 'social positive' vs 'social negative', (C) Women, 'social positive' vs 'non-social', (D) Men, 'social positive' vs 'non-social'.
